# Supplementary material for: Immunosenescence Profile Is Associated With Increased Susceptibility to Severe COVID‐19
Source: Aging Cell. 2025 May 19;24(7):e70077. doi: 10.1111/acel.70077 (PMC12266769; doi:10.1111/acel.70077)
Supplement: Supplementary file 1 — Figure S1. Viral load and serum levels of vitamin D in individuals with different clinical forms of COVID‐19. Figure S2. Global and individual production of plasma mediators in adult and elderly patients with either mild COVID‐19 or Flu‐like‐syndrome at different stages of infection. Figure S3. Gating strategy for the identification of the indicated T cell subsets in peripheral blood mononuclear cells (PBMCs). Figure S4. Frequencies of phenotypically characterized regulatory CD4+ FoxP3+ CD25+ and CD4+ Foxp3+ CD25+ PD‐1+ T cells in individuals with mild, moderate and severe COVID‐19. Figure S5. Sample selection flowchart (study design). [file ACEL-24-e70077-s001.pdf]

**a**

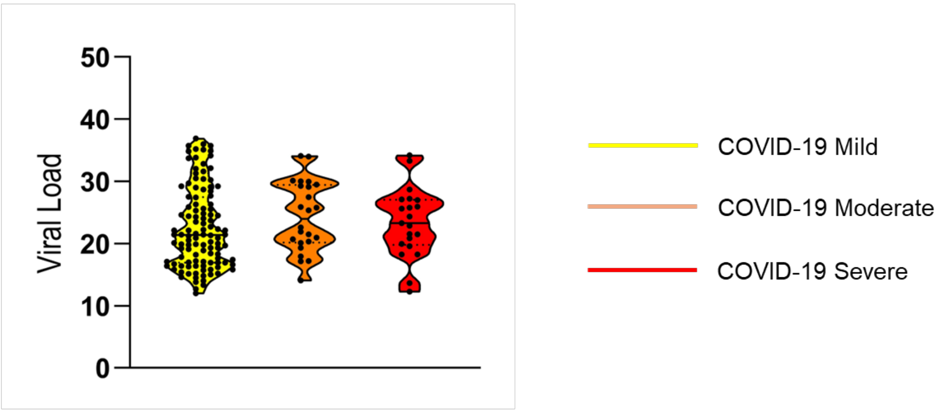


**b**

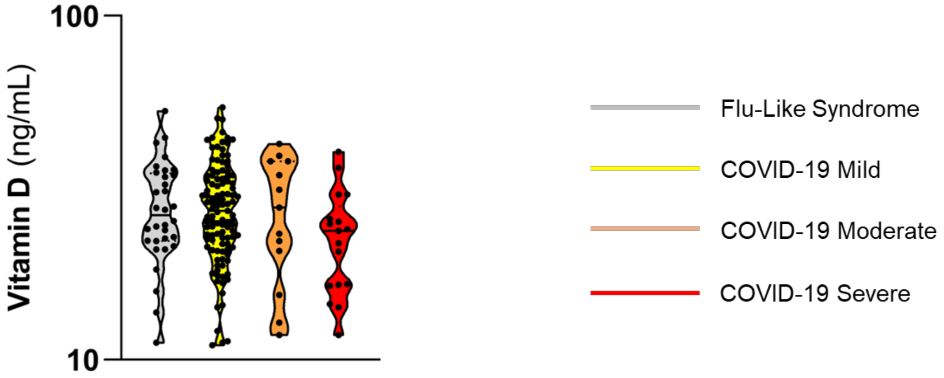


**Figure S1 – Viral load and serum levels of vitamin D in individuals with different clinical forms of COVID-19**. The cohort used for this analysis was composed by individuals from Belo Horizonte, São Paulo and Governador Valadares. (**a**) Viral load analysis included individuals with COVID-19 presenting different clinical forms: mild (n = 27), moderate (n = 6) and severe (n = 8). (**b**) Serum levels of vitamin D in individuals with different clinical forms of COVID-19 and in individuals with Flu-like syndrome (n = 34). The clinical forms considered were mild (n = 111), moderate (n = 14), and severe (n = 25). Samples were previously normalized, and outliers were excluded using the ROUT test. Mann-Whitney test was performed individually for each group. Lines were used to indicate groups that were compared and asterisks to represent statistically significant differences (p≤0.05, **p≤0.01, ***p≤0.001 or ****p<0.0001). Individuals in the groups were matched by sex as well as age and they were at the initial stage of infection (1-4 days of symptoms).

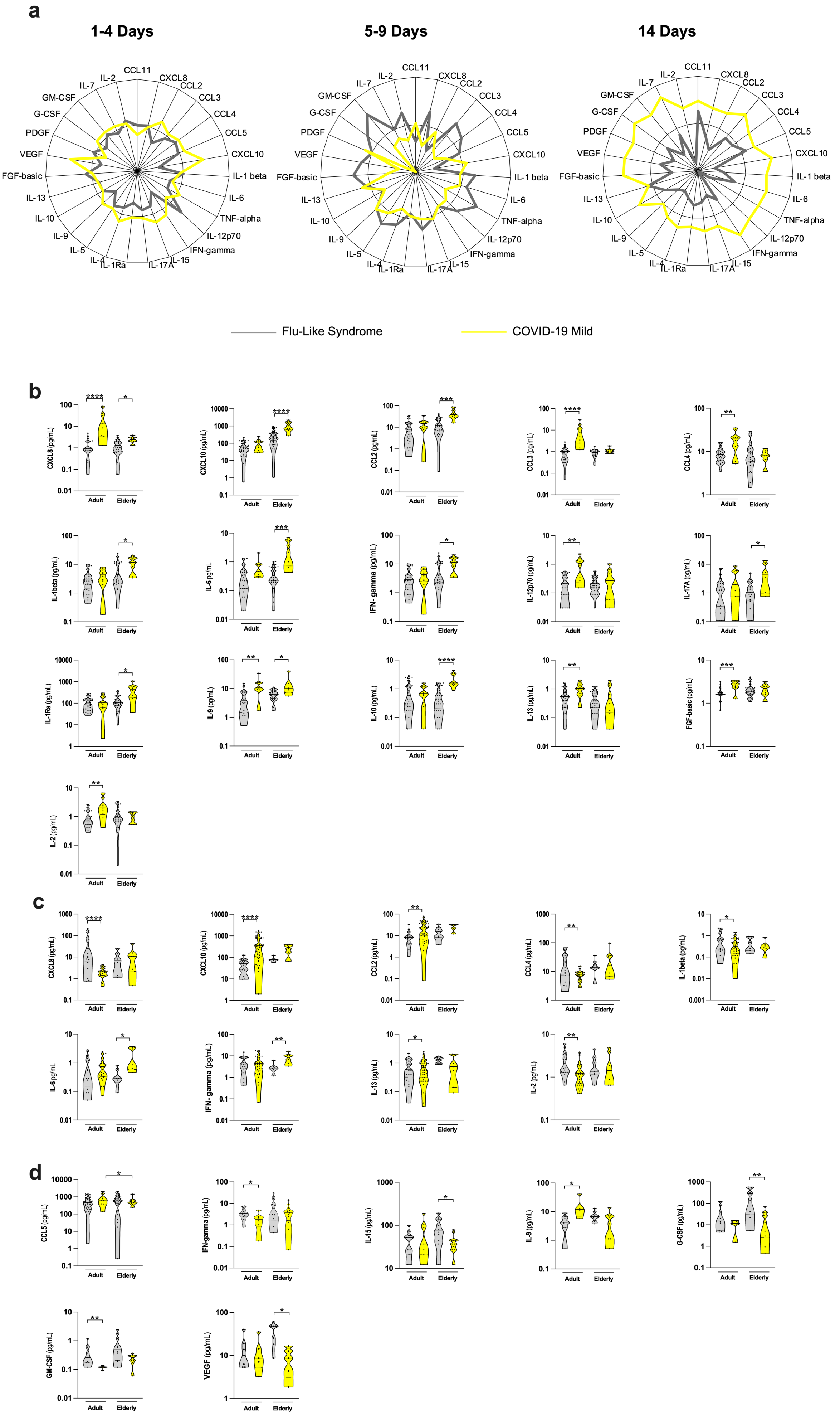
a
1-4 Days
5-9 Days
14 Days
CCL11
CXCL8
CCL2
CCL3
CCL4
CCL5
CXCL10
IL-1 beta
IL-6
TNF-alpha
IL-12p70
IFN-gamma
IL-15
IL-17A
IL-1Ra
IL-4
IL-5
IL-9
IL-10
IL-13
FGF-basic
VEGF
PDGF
G-CSF
GM-CSF
IL-7
IL-2
Flu-Like Syndrome
COVID-19 Mild
b
c
d
Adult
Elderly
CXCL8 (pg/mL)
CXCL10 (pg/mL)
CCL2 (pg/mL)
CCL3 (pg/mL)
CCL4 (pg/mL)
IL-1beta (pg/mL)
IL-6 (pg/mL)
IFN- gamma (pg/mL)
IL-12p70 (pg/mL)
IL-17A (pg/mL)
IL-1Ra (pg/mL)
IL-9 (pg/mL)
IL-10 (pg/mL)
IL-13 (pg/mL)
FGF-basic (pg/mL)
IL-2 (pg/mL)
CCL5 (pg/mL)
IFN-gamma (pg/mL)
IL-15 (pg/mL)
G-CSF (pg/mL)
GM-CSF (pg/mL)
VEGF (pg/mL)

**Figure S2 – Global and individual production of plasma mediators in adult and elderly patients with either mild COVID-19 or Flu-like-syndrome at different stages of infection. (a)** Radar chart with the frequency of high mediator producers among individuals from Belo Horizonte and São Paulo according to the days since symptom onset: 1 to 4 days with either Flu-like syndrome (FLS) (n = 50) or mild-COVID-19 (n = 41); 5 to 9 days with either Flu-like syndrome (FLS) (n = 37) or mild-COVID-19 (n = 53); 10 to 14 days with either Flu-like syndrome (FLS) (n = 14) or mild-COVID-19 (n = 15). Individuals in the groups were matched by sex and age. Differences in plasma mediator concentrations in adults and elderly with either FLS or mild COVID-19 at different time points of symptom onset: (**b)** 1 to 4 days (n = 103); **(c)** 5 to 9 days (n = 91); **(d)** 10 to 14 days (n = 30). Samples were previously normalized, and outliers were excluded using the ROUT test. Mann-Whitney test was performed individually for each group. Lines were used to indicate groups that were compared and asterisks to represent statistical significance (p≤0.05, **p≤0.01, ***p≤0.001 or ****p<0.0001).

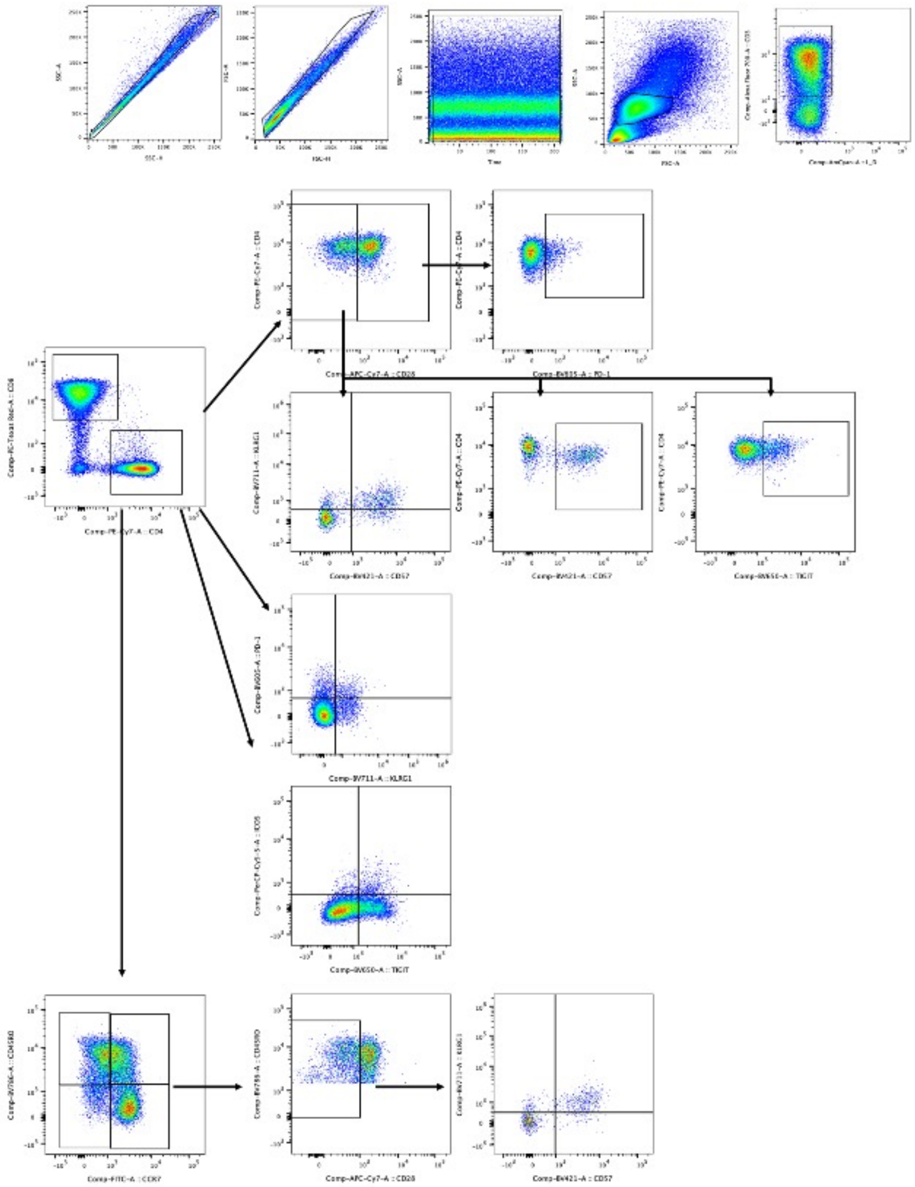

**Figure S3 – Gating strategy for the identification of the indicated T cell subsets in peripheral blood mononuclear cells (PBMCs).**

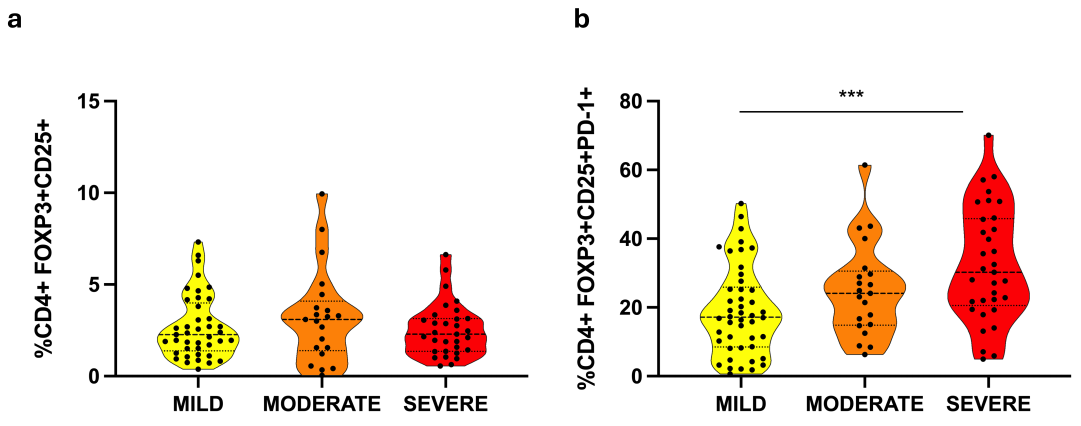


**Figure S4 – Frequencies of phenotypically characterized regulatory CD4+FoxP3+CD25+ and CD4+Foxp3+CD25+PD-1+ T cells in individuals with mild, moderate and severe COVID-19.** Individuals from Belo Horizonte and São Paulo with 1 to 7 days of symptoms were stratified into 3 groups classified according to the disease severity as mild (n= 46), moderate (n=21) and severe (n=33). Cells were gated using the CD4 marker. Surface expression of CD25 together with intracellular expression of Foxp3 were used to phenotypically identify regulatory T cells. PD-1 was used as a marker of exhaustion. Individuals in the groups were matched by sex and age. Frequencies of (**a**) CD4+Foxp3+CD25+ T cells and (**b**) CD4+Foxp3+CD25+PD-1+ are shown in the graphs. Samples were previously normalized, and outliers were excluded using the ROUT test. Mann-Whitney test was performed individually for each group. A line was used to indicate groups that were compared and the asterisk to represent statistical significance (***p≤0.001).

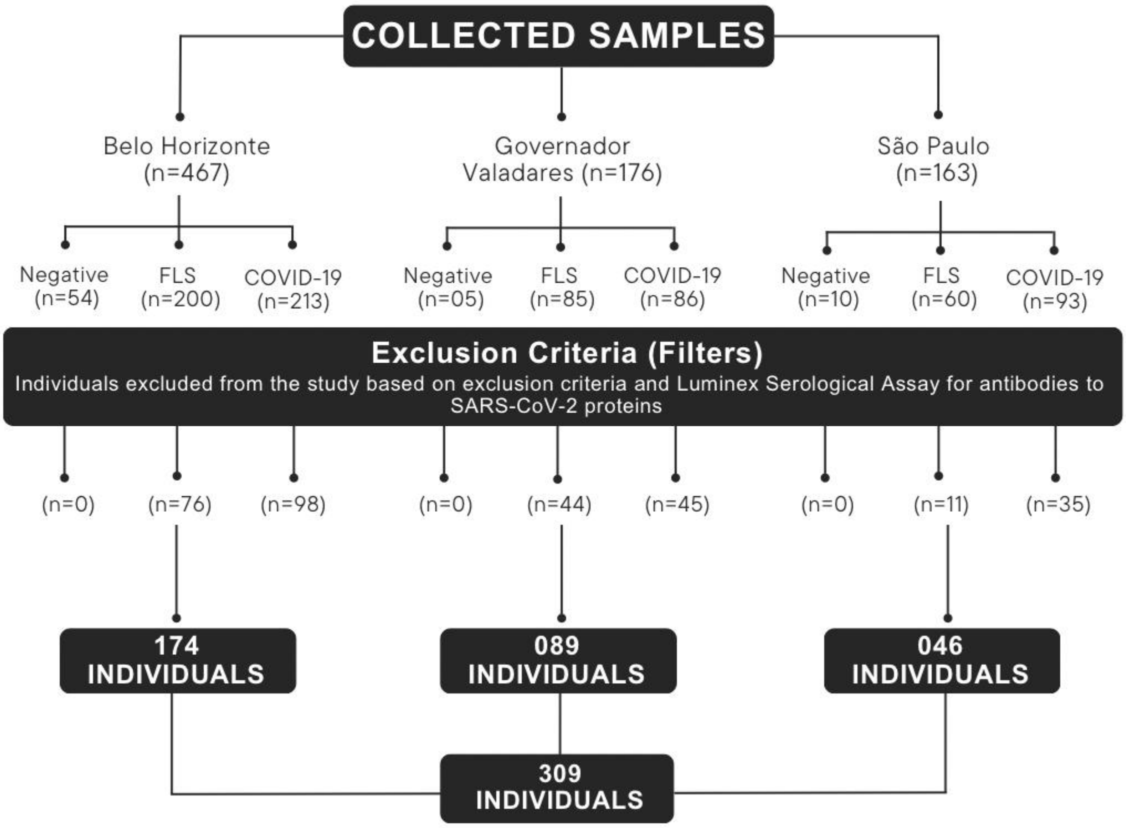


**Figure S5 – Sample selection flowchart (Study Design)**
