# Supplementary material for: Immunosenescence Profile Is Associated With Increased Susceptibility to Severe COVID‐19
Source: Aging Cell. 2025 May 19;24(7):e70077. doi: 10.1111/acel.70077 (PMC12266769; doi:10.1111/acel.70077)
Supplement: Supplementary file 2 — Table S1. WHO criteria for classification of the clinical groups. Table S2. Panel of monoclonal antibodies used for flow cytometry. Table S3. Raw data of immunoglobulin sequencing. Table S4. Summary of immunoglobulin (VH) sequences (repertoire analysis). [file ACEL-24-e70077-s002.pdf]

Table S1: Who criteria for the clinical groups

Patient State	*Descriptor*	*Score*
Uninfected	Uninfected; no viral RNA detected.	0
COVID-19 Mild	Assymptomatic; viral RNA detected.	1
	Symptomatic; Independent.	2
	Symptomatic; Assistance needed.	3
COVID-19 Moderate	Hospitalised; No oxygen therapy.	4
	Hospitalised; Oxygen by mask or nasal prongs.	5
COVID-19 Severe	Hospitalised; Oxygen by NIV or high flow.	6
	Intubation and mechanical ventilation, $pO_2/FiO_2 \geq 150$ or $SpO_2/FIO_2 \geq 200$).	7
	Mechanical ventilation, $pO_2/FiO_2 < 200$) or vasopressor.	8
	Mechanical ventilation, $pO_2/FiO_2 < 150$ and vasopressor, dyalysis or ECMO.	9
	Dead.	10

Table S2: Panel of monoclonal Antibodies for flow cytometry

	Antibody	Channel	Dilution Used	Catalog Number	Clone
T Cell Panel	CD4	Pe Cy 7	1:400	557852	SK3
	CD8	PE-Dazzle 594	1:1200	562282	RPA-T8
	CD25	PE	1:20	555432	M-A251
	CD278 (ICOS)	Percp Cy 5.5	1:20	562833	DX29
	CD57	BV 421	1:800	563896	NK-1
	CD279 (PD-1)	BV 605	1:40	563245	EH12.1
	TIGIT	BV 650	1:160	741182	FAB7898G
	CD28	APC H7	1:20	561368	CD28.2
	CD3	AF 700	1:320	317340	OKT3
	CCR7	FITC	1:40	353216	G943H7
	KLRG-1	BV 711	1:40	138427	2F1/KLRG1
	FOXP3	APC Cy7	1:20	21276106	3G3
	CD45RO	BV 785	1:80	304234	UCHL1
NK Cell Panel	CD3	AF 700	1:320	317340	OKT3
	CD16	PE Cy 5.5	1:100	555408	3G8
	CD25	PE	1:20	555432	M-A251
	CD56	PE Cy 7	1:200	340723	NCAM 16.2
	CD57	BV 421	1:640	563896	NK-1
	CD69	APC Cy7	1:20	557756	FN50
	CD 279 (PD-1)	BV 605	1:40	563245	EH12.1
	NKG2D	APC	1:20	558071	1D11
B Cell Panel	CD24	FITC	1:160	555427	ML5
	CD 279 (PD-1)	BV 605	1:40	563245	EH12.1
	CD38	PE	1:20	555460	HIT2
	CD5	Percp Cy 5.5	1:80	341089	L17F12
	CD19	PE Cy 7	1:640	557835	SJ25C1
	IgD	AF 700	1:40	561302	IA6-2
	CD138	APC	1:20	347193	MI15
	CD27	APC Cy7	1:20	560222	M-T271
	CD20	BV 650	1:800	563780	2H7

Table S3: Raw Data of Immunoglobulin Sequencing

City	Clinical Outcome	Sample	Raw read	Pre-processed reads*	Annotated reads	Clones
**Belo Horizonte**	**Control**	**A04**	406,894	246,973	244,359	16,300
**Belo Horizonte**	**Control**	**A20**	618,809	420,539	418,354	12,662
**Belo Horizonte**	**Control**	**A24**	1,362,447	411,169	403,966	9,567
**Belo Horizonte**	**Control**	**A65**	555,155	348,297	344,143	15,331
**Belo Horizonte**	**Control**	**A66**	761,554	478,945	472,011	17,086
**Belo Horizonte**	**Control**	**A67**	546,518	354,867	350,617	31,830
**Governador Valadares**	**Mild**	**GV106**	537,812	255,051	199,460	6,956
**Governador Valadares**	**Mild**	**GV144**	349,768	215,476	193,437	9,710
**Governador Valadares**	**Mild**	**GV146**	1,450,041	398,780	395,181	9,087
**Governador Valadares**	**Mild**	**GV43**	906,435	279,639	271,681	13,703
**Governador Valadares**	**Mild**	**GV47**	544,092	202,199	159,930	6,907
**Governador Valadares**	**Mild**	**GV50**	1,332,292	299,198	236,633	8,494
**Governador Valadares**	**Mild**	**GV51**	1,477,827	306,737	295,683	8,186
**Governador Valadares**	**Mild**	**GV54**	628,211	264,316	245,602	8,940
**Governador Valadares**	**Mild**	**GV68**	558,832	344,804	338,562	13,168
**Governador Valadares**	**Mild**	**GV92**	487,172	203,943	130,060	3,053
**Belo Horizonte**	**Mild**	**ID094**	620,176	410,089	408,93	7,021
**Belo Horizonte**	**Mild**	**ID117**	540,888	341,650	340,256	13,239
**Belo Horizonte**	**Mild**	**ID124**	632,642	427,965	422,618	36,806
**Belo Horizonte**	**Mild**	**ID131**	536,784	311,349	304,709	9,865
**Belo Horizonte**	**Mild**	**ID132**	436,757	264,768	262,704	32,069
**Belo Horizonte**	**Hospitalized**	**ID141**	412,936	235,970	233,636	10,807
**Belo Horizonte**	**Hospitalized**	**ID143**	341,073	232,296	231,034	11,192
**Belo Horizonte**	**Hospitalized**	**ID144**	510,649	347,086	337,768	9,042
**Belo Horizonte**	**Mild**	**ID155**	557,750	323,370	311,478	14,827
**Belo Horizonte**	**Hospitalized**	**ID187**	563,901	295,370	284,909	26,310
**Belo Horizonte**	**Hospitalized**	**ID195**	406167	233,411	229,688	6,807
**Belo Horizonte**	**Hospitalized**	**ID226**	1,525,890	333,602	330,351	15,422
**Belo Horizonte**	**Mild**	**ID240**	407,527	236,377	229,633	8,234
**Belo Horizonte**	**Mild**	**ID244**	430,185	260,499	256,455	18,387
**Belo Horizonte**	**Hospitalized**	**ID248**	405,371	277,585	272,233	18,185
**Belo Horizonte**	**Hospitalized**	**ID268**	285,341	180,243	179,320	30,896
**Belo Horizonte**	**Hospitalized**	**ID310**	638,973	418,567	417,669	14,627
**Belo Horizonte**	**Hospitalized**	**ID375**	638,037	379,779	374,808	69,737
**São Paulo**	**Hospitalized**	**SP10**	386,469	205,262	194,655	23,650
**São Paulo**	**Hospitalized**	**SP114**	429,407	243,112	238,327	9,007
**São Paulo**	**Hospitalized**	**SP138**	790,021	550,456	548,060	23,304
**São Paulo**	**Mild**	**SP14**	521,830	324,709	321,397	41,497
**São Paulo**	**Hospitalized**	**SP20**	639,344	366,906	363,329	12,671

*Reads after steps 1, 2, and 3 of pre-processing.

Table S4: Summary of VIg sequences (repertoire)

	NAME	SEQUENCE (5' TO 3')
**1**	VH1-fwd	**TCGTCGGCAGCGTCAGATGTGTATAAGAGACAG**CAGGTCCAGCTKGTRCAGTCTGG
**2**	VH157-fwd	**TCGTCGGCAGCGTCAGATGTGTATAAGAGACAG**CAGGTGCAGCTGGTGSARTCTGG
**3**	VH2-fwd	**TCGTCGGCAGCGTCAGATGTGTATAAGAGACAG**CAGRTCACCTTGAAGGAGTCTG
**4**	VH3-fwd	**TCGTCGGCAGCGTCAGATGTGTATAAGAGACAG**GAGGTGCAGCTGKTGGAGWCY
**5**	VH4-fwd	**TCGTCGGCAGCGTCAGATGTGTATAAGAGACAG**CAGGTGCAGCTGCAGGAGTCSG
**6**	VH4-DP63-fwd	**TCGTCGGCAGCGTCAGATGTGTATAAGAGACAG**CAGGTGCAGCTACAGCAGTGGG
**7**	VH6-fwd	**TCGTCGGCAGCGTCAGATGTGTATAAGAGACAG**CAGGTACAGCTGCAGCAGTCA
**8**	VH3N-fwd	**TCGTCGGCAGCGTCAGATGTGTATAAGAGACAG**TCAACACAACGGTTCCCAGTTA
**9**	IgG-rev	**GTCTCGTGGGCTCGGAGATGTGTATAAGAGACAG**AGGGYGCCAGGGGGAAGAC
**10**	IgA-rev	**GTCTCGTGGGCTCGGAGATGTGTATAAGAGACAG**CGGGAAGACCTTGGGGCTGG

The bold sequences constitute elements of the Illumina adaptor sequences. The design of the annealing region in primers was based on MacDaniel et al. (2016).

Table S5: Summary of Biomarkers of Network Analysis

ID	BIOMARKER
**1L**	CCL11 (Luminex)
**2L**	CXCL8 (Luminex)
**3L**	CCL2 (Luminex)
**4L**	CCL3 (Luminex)
**5L**	CCL4 (Luminex)
**6L**	CCL5 (Luminex)
**7L**	CXCL10 (Luminex)
**8PI**	IL-1β (Luminex)
**9PI**	IL-6 (Luminex)
**10PI**	TNF (Luminex)
**11PI**	IL-12(p70) (Luminex)
**12PI**	IFN-γ (Luminex)
**13PI**	IL-15 (Luminex)
**14PI**	17-A (Luminex)
**15R**	IL-1Ra (Luminex)
**16R**	IL-4 (Luminex)
**17R**	IL-5 (Luminex)
**18R**	IL-9 (Luminex)
**19R**	IL-10 (Luminex)
**20R**	IL-13 (Luminex)
**21GF**	FGF-basic (Luminex)
**22GF**	VEGF (Luminex)
**23GF**	PDGF-BB (Luminex)
**24GF**	G-CSF (Luminex)
**25GF**	GM-CSF (Luminex)
**26GF**	IL-7 (Luminex)
**27GF**	IL-2 (Luminex)
**1C**	CCL2 (CBA)
**2C**	CCL5 (CBA)
**3C**	CXCL8 (CBA)
**4C**	CXCL9 (CBA)
**5C**	CXCL10 (CBA)
**AGE**	Age
**OUTCOME**	Outcome
**1T**	Total Cells
**2T**	Total Live Cells
**3T**	Lymphocytes
**4T**	Lymphocytes Live
**5T**	Lymphocytes CD3+
**6T**	Lymphocytes CD3+CD4+
**7T**	Lymphocytes CD3+CD4+CD25(High)
**8T**	Lymphocytes CD3+CD4+CD25(Low)
**9T**	Lymphocytes CD3+CD4+CD25+FOXP3+
**10T**	Lymphocytes CD3+CD4+CD25+FOXP3+PD1+
**11T**	Lymphocytes CD3+CD4+CD28-CD4+
**12T**	Lymphocytes CD3+CD4+CD28-CD4+/CD4+CD28-CD57+
**13T**	Lymphocytes CD3+CD4+CD28-CD4+/CD4+CD28-KLRG1+
**14T**	Lymphocytes CD3+CD4+CD28-CD4+/CD4+CD28-PD-1+
**15T**	Lymphocytes CD3+CD4+CD28-CD4+/CD4+CD28-TIGIT+
**16T**	Lymphocytes CD3+CD4+CD28-CD4+/Q1:CD57-KLRG1+
**17T**	Lymphocytes CD3+CD4+CD28-CD4+/Q2:CD57+KLRG1+
**18T**	Lymphocytes CD3+CD4+CD28-CD4+/Q3:CD57+KLRG1-
**19T**	Lymphocytes CD3+CD4+CD28-CD4+/Q4:CD57-KLRG1-
**20T**	Lymphocytes CD3+CD4+/CD57+CD4+
**21T**	Lymphocytes CD3+CD4+/CM CD4+
**22T**	Lymphocytes CD3+CD4+/EFF CD4+
**23T**	Lymphocytes CD3+CD4+/EFF CD4+/ CD4+ EFF CD28-
**24T**	Lymphocytes CD3+CD4+/EFF CD4+/ CD4+ EFF CD28-/ Q1: CD57-KLRG1+

**25T**	Lymphocytes CD3+CD4+/EFF CD4+/ CD4+ EFF CD28-/ Q2: CD57+KLRG1+
**26T**	Lymphocytes CD3+CD4+/EFF CD4+/ CD4+ EFF CD28-/ Q4: CD57+KLRG1-
**27T**	Lymphocytes CD3+CD4+/EFF CD4+/ CD4+ EFF CD28-/ Q4: CD57-KLRG1-
**28T**	Lymphocytes CD3+CD4+/EM CD4+
**29T**	Lymphocytes CD3+CD4+/EM CD4+/ EM CD28- CD45RO+
**30T**	Lymphocytes CD3+CD4+/EM CD4+/ EM CD28- CD45RO+/ CD4+ EM CD28-CD57+
**31T**	Lymphocytes CD3+CD4+/EM CD4+/ EM CD28- CD45RO+/ CD4+ EM CD28-KLRG1+
**32T**	Lymphocytes CD3+CD4+/EM CD4+/ EM CD28- CD45RO+/ CD4+ EM CD28- PD-1+
**33T**	Lymphocytes CD3+CD4+/EM CD4+/ EM CD28- CD45RO+/ Q1: CD57-KLRG1+
**34T**	Lymphocytes CD3+CD4+/EM CD4+/ EM CD28- CD45RO+/ Q2: CD57+KLRG1+
**35T**	Lymphocytes CD3+CD4+/EM CD4+/ EM CD28- CD45RO+/ Q3: CD57+KLRG1-
**36T**	Lymphocytes CD3+CD4+/EM CD4+/ EM CD28- CD45RO+/ Q4: CD57-KLRG1-
**37T**	Lymphocytes CD3+CD4+/EM CD4+/ EM CD28- CD45RO+/ Q5: CD57-ICOS+
**38T**	Lymphocytes CD3+CD4+/EM CD4+/ EM CD28- CD45RO+/ Q6: CD57+ICOS+
**39T**	Lymphocytes CD3+CD4+/EM CD4+/ EM CD28- CD45RO+/ Q7: CD57+ICOS-
**40T**	Lymphocytes CD3+CD4+/EM CD4+/ EM CD28- CD45RO+/ Q8: CD57-ICOS-
**41T**	Lymphocytes CD3+CD4+/ KLRG1+CD4+
**42T**	Lymphocytes CD3+CD4+/ NAÏVE CD4+
**43T**	Lymphocytes CD3+CD4+/ PD-1+CD4+
**44T**	Lymphocytes CD3+CD4+/ Q1: CD57- PD-1+
**45T**	Lymphocytes CD3+CD4+/ Q2: CD57+ PD-1+
**46T**	Lymphocytes CD3+CD4+/ Q3: CD57+ PD-1-
**47T**	Lymphocytes CD3+CD4+/ Q4: CD57- PD-1-
**48T**	Lymphocytes CD3+CD4+/ Q5: CD57- TIGIT+
**49T**	Lymphocytes CD3+CD4+/ Q6: CD57+ TIGIT+
**50T**	Lymphocytes CD3+CD4+/ Q7: CD57+ TIGIT-
**51T**	Lymphocytes CD3+CD4+/ Q8: CD57- TIGIT-
**52T**	Lymphocytes CD3+CD4+/ Q9: KLRG1- PD-1+
**53T**	Lymphocytes CD3+CD4+/ Q10: KLRG1+ PD-1+
**54T**	Lymphocytes CD3+CD4+/ Q11: KLRG1+ PD-1-
**55T**	Lymphocytes CD3+CD4+/ Q12: KLRG1- PD-1-
**56T**	Lymphocytes CD3+CD4+/ TIGIT+ CD4+
**57T**	Lymphocytes CD3+CD8+
**58T**	Lymphocytes CD3+CD8+/ CD8+CD28+
**59T**	Lymphocytes CD3+CD8+/ CD8+CD28+/ CD8+CD28+CD57+
**60T**	Lymphocytes CD3+CD8+/ CD8+CD28+/ CD8+CD28+ICOS+
**61T**	Lymphocytes CD3+CD8+/ CD8+CD28+/ CD8+CD28+KLRG1+
**62T**	Lymphocytes CD3+CD8+/ CD8+CD28+/ CD8+CD28+PD-1+
**63T**	Lymphocytes CD3+CD8+/ CD8+CD28+/ Q1:CD57- KLRG1+
**64T**	Lymphocytes CD3+CD8+/ CD8+CD28+/ Q2:CD57+ KLRG1+
**65T**	Lymphocytes CD3+CD8+/ CD8+CD28+/ Q3:CD57+ KLRG1-
**66T**	Lymphocytes CD3+CD8+/ CD8+CD28+/ Q4:CD57- KLRG1-
**67T**	Lymphocytes CD3+CD8+/ CD8+CD28+/ Q5:CD57- PD-1+
**68T**	Lymphocytes CD3+CD8+/ CD8+CD28+/ Q6:CD57+ PD-1+
**69T**	Lymphocytes CD3+CD8+/ CD8+CD28+/ Q7:CD57+ PD-1-
**70T**	Lymphocytes CD3+CD8+/ CD8+CD28+/ Q8:CD57- PD-1-
**71T**	Lymphocytes CD3+CD8+/ CD8+CD57+
**72T**	Lymphocytes CD3+CD8+/ CD8+ICOS+
**73T**	Lymphocytes CD3+CD8+/ CD8+KLRG1+
**74T**	Lymphocytes CD3+CD8+/ CD8+NAÏVE
**75T**	Lymphocytes CD3+CD8+/ CD8+PD-1+
**76T**	Lymphocytes CD3+CD8+/ CD8+TIGIT+
**77T**	Lymphocytes CD3+CD8+/ CD28-CD8+
**78T**	Lymphocytes CD3+CD8+/ CD28-CD8+/ CD8+CD28-CD57+
**79T**	Lymphocytes CD3+CD8+/ CD28-CD8+/ CD8+CD28-ICOS+
**80T**	Lymphocytes CD3+CD8+/ CD28-CD8+/ CD8+CD28-KLRG1+
**81T**	Lymphocytes CD3+CD8+/ CD28-CD8+/ CD8+CD28-PD-1+
**82T**	Lymphocytes CD3+CD8+/ CD28-CD8+/ CD8+CD28-TIGIT+
**83T**	Lymphocytes CD3+CD8+/ CD28-CD8+/ Q1: CD57- KLRG1+
**84T**	Lymphocytes CD3+CD8+/ CD28-CD8+/ Q2: CD57+ KLRG1+
**85T**	Lymphocytes CD3+CD8+/ CD28-CD8+/ Q3: CD57+ KLRG1-

**86T**	Lymphocytes CD3+CD8+/ CD28-CD8+/ Q4: CD57- KLRG1-
**87T**	Lymphocytes CD3+CD8+/ CM CD8+
**88T**	Lymphocytes CD3+CD8+/ EFF CD8+
**89T**	Lymphocytes CD3+CD8+/ EFF CD8+/ CD8+ EFF CD28-
**90T**	Lymphocytes CD3+CD8+/ EFF CD8+/ CD8+ EFF CD28-/ CD8+ EFF CD28-CD57+
**91T**	Lymphocytes CD3+CD8+/ EFF CD8+/ CD8+ EFF CD28-/ CD8+ EFF CD28-KLRG1+
**92T**	Lymphocytes CD3+CD8+/ EFF CD8+/ CD8+ EFF CD28-/ CD8+ EFF CD28-PD-1+
**93T**	Lymphocytes CD3+CD8+/ EFF CD8+/ CD8+ EFF CD28-/ CD8+ EFF CD28-TIGIT+
**94T**	Lymphocytes CD3+CD8+/ EFF CD8+/ CD8+ EFF CD28-/ Q2: CD57+ KLRG1+
**95T**	Lymphocytes CD3+CD8+/ EFF CD8+/ CD8+ EFF CD28-/ Q3: CD57+ KLRG1-
**96T**	Lymphocytes CD3+CD8+/ EFF CD8+/ CD8+ EFF CD28-/ Q4: CD57- KLRG1-
**97T**	Lymphocytes CD3+CD8+/ EFF CD8+/ CD8+ EFF CD28-/ Q5: CD57- PD-1+
**98T**	Lymphocytes CD3+CD8+/ EFF CD8+/ CD8+ EFF CD28-/ Q6: CD57+ PD-1+
**99T**	Lymphocytes CD3+CD8+/ EFF CD8+/ CD8+ EFF CD28-/ Q7: CD57+ PD-1-
**100T**	Lymphocytes CD3+CD8+/ EFF CD8+/ CD8+ EFF CD28-/ Q8: CD57- PD-1-
**101T**	Lymphocytes CD3+CD8+/ EFF CD8+/ CD8+ EFF CD28-/ Q9: CD57- ICOS+
**102T**	Lymphocytes CD3+CD8+/ EFF CD8+/ CD8+ EFF CD28-/ Q10: CD57+ ICOS+
**103T**	Lymphocytes CD3+CD8+/ EFF CD8+/ CD8+ EFF CD28-/ Q11: CD57+ ICOS-
**104T**	Lymphocytes CD3+CD8+/ EFF CD8+/ CD8+ EFF CD28-/ Q12: CD57- ICOS-
**105T**	Lymphocytes CD3+CD8+/ EFF CD8+/ CD8+ EFF CD28-/ Q1: CD57- KLRG1+
**106T**	Lymphocytes CD3+CD8+/ EFF CD8+/ CD8+ EFF CD57+
**107T**	Lymphocytes CD3+CD8+/ EFF CD8+/ CD8+ EFF PD-1+
**108T**	Lymphocytes CD3+CD8+/ EFF CD8+/ CD8+ EFF TIGIT+
**109T**	Lymphocytes CD3+CD8+/ EFF CD8+/ Q1: CD57- TIGIT+
**110T**	Lymphocytes CD3+CD8+/ EFF CD8+/ Q2: CD57+ TIGIT+
**111T**	Lymphocytes CD3+CD8+/ EFF CD8+/ Q3: CD57+ TIGIT-
**112T**	Lymphocytes CD3+CD8+/ EFF CD8+/ Q4: CD57- TIGIT-
**113T**	Lymphocytes CD3+CD8+/ EM CD8+
**114T**	Lymphocytes CD3+CD8+/ EM CD8+/ CD8+ EM CD57+
**115T**	Lymphocytes CD3+CD8+/ EM CD8+/ CD8+ EM ICOS+
**116T**	Lymphocytes CD3+CD8+/ EM CD8+/ CD8+ EM KLRG1+
**117T**	Lymphocytes CD3+CD8+/ EM CD8+/ CD8+ EM PD-1+
**118T**	Lymphocytes CD3+CD8+/ EM CD8+/ CD8+ EM TIGIT+
**119T**	Lymphocytes CD3+CD8+/ EM CD8+/ CD28- CD45RO+
**120T**	Lymphocytes CD3+CD8+/ EM CD8+/ CD28- CD45RO+/ CD8+ EM CD28-CD57+
**121T**	Lymphocytes CD3+CD8+/ EM CD8+/ CD28- CD45RO+/ CD8+ EM CD28-KLRG1+
**122T**	Lymphocytes CD3+CD8+/ EM CD8+/ CD28- CD45RO+/ CD8+ EM CD28-PD-1+
**123T**	Lymphocytes CD3+CD8+/ EM CD8+/ CD28- CD45RO+/ Q2: CD57+ KLRG1+
**124T**	Lymphocytes CD3+CD8+/ EM CD8+/ CD28- CD45RO+/ Q3: CD57+ KLRG1-
**125T**	Lymphocytes CD3+CD8+/ EM CD8+/ CD28- CD45RO+/ Q4: CD57- KLRG1-
**126T**	Lymphocytes CD3+CD8+/ EM CD8+/ CD28- CD45RO+/ Q5: CD57- PD-1+
**127T**	Lymphocytes CD3+CD8+/ EM CD8+/ CD28- CD45RO+/ Q6: CD57+ PD-1+
**128T**	Lymphocytes CD3+CD8+/ EM CD8+/ CD28- CD45RO+/ Q8: CD57- PD-1-
**129T**	Lymphocytes CD3+CD8+/ EM CD8+/ CD28- CD45RO+/ Q9: ICOS- TIGIT+
**130T**	Lymphocytes CD3+CD8+/ EM CD8+/ CD28- CD45RO+/ Q10: ICOS+ TIGIT+
**131T**	Lymphocytes CD3+CD8+/ EM CD8+/ CD28- CD45RO+/ Q11: ICOS+ TIGIT-
**132T**	Lymphocytes CD3+CD8+/ EM CD8+/ CD28- CD45RO+/ Q12: ICOS- TIGIT-
**133T**	Lymphocytes CD3+CD8+/ EM CD8+/ CD28- CD45RO+/ Q13: CD57- TIGIT+
**134T**	Lymphocytes CD3+CD8+/ EM CD8+/ CD28- CD45RO+/ Q14: CD57+ TIGIT+
**135T**	Lymphocytes CD3+CD8+/ EM CD8+/ CD28- CD45RO+/ Q15: CD57+ TIGIT-
**136T**	Lymphocytes CD3+CD8+/ EM CD8+/ CD28- CD45RO+/ Q16: CD57- KLRG1-
**137T**	Lymphocytes CD3+CD8+/ EM CD8+/ CD28- CD45RO+/ Q1: CD57- KLRG1+
**138T**	Lymphocytes CD3+CD8+/ EM CD8+/ Q1: CD57- PD-1+
**139T**	Lymphocytes CD3+CD8+/ EM CD8+/ Q2: CD57+ PD-1+
**140T**	Lymphocytes CD3+CD8+/ EM CD8+/ Q3: CD57+ PD-1-
**141T**	Lymphocytes CD3+CD8+/ EM CD8+/ Q4: CD57- PD-1-
**HRV**	Horvath's clock
**TL**	Telomere Length's clock
**BLU**	BLUP clock
**SHM**	Shannon's Entropy

Summary representing the code for each biomarker used in the network analysis and the corresponding mediator.
